# Generation and discrimination of autism MRI images based on autoencoder

**DOI:** 10.3389/fpsyt.2024.1395243

**Published:** 2024-10-14

**Authors:** Yuxin Shi, Yongli Gong, Yurong Guan, Jiawei Tang

**Affiliations:** ^1^ Computer School, Hubei Polytechnic University, Huangshi, China; ^2^ College of Computer Science, Huanggang Normal University, Huanggang, China

**Keywords:** deep autoencoder, sMRI, image generation, image classification, ASD

## Abstract

This study aims to explore an autoencoder-based method for generating brain MRI images of patients with Autism Spectrum Disorder (ASD) and non-ASD individuals, and to discriminate ASD based on the generated images. Initially, we introduce the research background of ASD and related work, as well as the application of deep learning in the field of medical imaging. Subsequently, we detail the architecture and training process of the proposed autoencoder model, and present the results of generating MRI images for ASD and non-ASD patients. Following this, we designed an ASD classifier based on the generated images and elucidated its structure and training methods. Finally, through analysis and discussion of experimental results, we validated the effectiveness of the proposed method and explored future research directions and potential clinical applications. This research offers new insights and methodologies for addressing challenges in ASD studies using deep learning technology, potentially contributing to the automated diagnosis and research of ASD.

## 1 Introduction

Autism Spectrum Disorder (ASD) is a neurodevelopmental disorder characterized by deficits in social interaction and communication, as well as restricted behaviors. Over the past decades, with the advancement of medical imaging technology, researchers have begun utilizing Magnetic Resonance Imaging (MRI) to study the differences in brain structure and functional connectivity in individuals with ASD. However, due to the high heterogeneity and complexity of ASD, relying solely on expert manual analysis of MRI images has its limitations. Thus, the use of deep learning technologies for the analysis and diagnosis of ASD has become increasingly important.

As machine learning and deep learning continue to evolve, significant progress has been made in the analysis and diagnosis of ASD. For instance, Support Vector Machines (SVM) have been widely used and studied in brain medical image classification. Early research by Katuwal et al. (1) explored SVM classifiers using MRI data to detect ASD, demonstrating that classification accuracy increases with the severity of autism. Beyond SVM, other machine learning methods have also achieved notable successes in ASD analysis and diagnostics. Bhaumik et al. (2) used Partial Least Squares Regression (PLS) based on features obtained from 42 bilateral Brodmann areas to distinguish between ASD patients and healthy controls. Donato et al. (3) employed Bayesian methods for reverse inference studies, finding probabilistic evidence of gray matter changes selective to ASD in specific brain regions.

Compared to machine learning, deep learning can learn high-level features from data without the need for expert identification (4). The applications of deep learning in ASD diagnostics and analysis can be broadly divided into three categories: methods based on Convolutional Neural Networks (CNNs), Generative Adversarial Networks (GANs), and autoencoders. CNN methods often employ various CNN variants or increase the depth of CNNs to handle the high dimensionality of MRI data, making them suitable for image classification tasks in deep learning. Zhao et al. (5) proposed a 3D CNN framework to capture the overlapping spatial brain network patterns between ASD and healthy controls, proving it to be effective. Dolz et al. (6) used a 3D CNN ensemble model with internally annotated images for 3D segmentation of infant T1-weighted and T2-weighted MRI images, achieving notable performance. Recently, Yin et al. (7) combined autoencoders with Deep Neural Networks (DNN), achieving 82.4% AUC and 79.2% accuracy. While CNNs effectively leverage spatial structural information across the entire brain, they have limitations in handling spatial coordinate transformation-related issues (8). Therefore, in ASD image generation tasks, integrating CNNs with other models is preferred.

GANs, as generative models, utilize game theory methods to generate images, thereby enhancing ASD datasets. Yang et al. (9) successfully improved classifier performance by using FC-GAN to augment ASD datasets. Recently, Devika et al. (10) employed a GAN-based encoder-decoder framework using consecutive sMRI slices as input, where the error between the reconstructed and actual adjacent slice stacks was used to identify ASD samples as outliers, yielding effective results. GANs learn the distribution of real data to generate lifelike images, and their generators can capture multiple data patterns, adding diversity to the generated images. However, training GAN models can be challenging due to the need to achieve Nash equilibrium (11).

Meanwhile, the accuracy of MRI image diagnostics depends on the quality of the images, which is often compromised by noise and artifacts (12). Thus, MRI image generation methods that also assist in noise reduction can significantly enhance image quality. For image denoising, autoencoders are generally more suitable than GANs.

For high-dimensional data image reconstruction (13) and noise removal (14), autoencoders are an excellent choice. As a deep learning model, autoencoders learn data features to reconstruct data. Their advantages include the ability to learn data representations that capture essential features of the data while minimizing redundancy, reducing the dimensionality of the data to make it more manageable, and removing noise from images to enhance clarity and accuracy (15). Autoencoders minimize the difference between input and reconstructed output during training, attempting to remove noise or unnecessary information from input images, thus typically providing a more stable model training process. Given the high dimensionality of neuroimaging data and the complexity involved in manually creating features from such data, the applications of autoencoders in brain disease research are of significant relevance (16).

Autoencoders have been used for MRI data generation and reconstruction, yielding positive research results. For example, Pinaya et al. (17) first trained a deep autoencoder model using data from healthy subjects, then estimated the overall and regional neuroanatomical deviations in ASD patients using the ASD dataset. This model provides a flexible framework for assessing overall and regional neuroanatomical deviations in neuropsychiatric populations. Li et al. (18), based on transfer learning techniques, proposed a novel deep neural network framework aimed at improving the ASD classification problem in situations with limited training samples. This framework first trained an SSAE using healthy subject data, which was then transferred to a new classification task for target subjects to achieve more accurate classification. Eslami et al. (19) proposed an Auto-ASD-Network model that combines the advantages of autoencoders and SVM, aimed at classifying ASD patients from healthy individuals using fMRI data, significantly enhancing classification results. Most recently, Mostafa (20) proposed using a CAE model to reconstruct images of ASD and healthy control groups. This model first processes 3D T1 images through slice analysis and classification, then inputs the slice images into an autoencoder for image reconstruction, using various similarity metrics to measure the resemblance between actual and reconstructed images, achieving high accuracy. These studies show that autoencoders can extract key features from MRI data, aiding medical experts in better understanding the brain structures and functions of ASD patients, providing support for early diagnosis and personalized treatment of the disorder. These studies demonstrate that, within ASD research, autoencoders are capable of extracting crucial features from MRI data, aiding medical experts in better understanding the brain structure and function of ASD patients. This facilitates early diagnosis and personalized treatment of the disorder.

Given this, we will detail the architecture and training process of the proposed autoencoder model, as well as how to use this model to generate MRI images of ASD and non-ASD patients. Next, we will introduce the structure and training methods of our designed ASD classifier, and describe in detail how to use the generated MRI images for ASD discrimination. Finally, we will analyze the experimental results and discuss future research directions and potential clinical applications. Specific research contributions include:

1. Autoencoder-generated MRI images of ASD and non-ASD patients: By designing and training the autoencoder model, this paper successfully generates MRI images for ASD and non-ASD patients. This method not only expands the dataset and increases sample diversity but also helps understand the characteristic brain structure features and differences in ASD patients. This is significant for ASD research in situations with limited sample sizes and high data heterogeneity.
2. ASD discrimination method based on generated images: Using the generated MRI images, this paper proposes a novel ASD classifier for discriminating patients with ASD. Compared to traditional methods based on original MRI images, this image generation-based approach offers more universality and effectively addresses issues of data sparsity and label imbalance. By combining autoencoder generation and deep learning classification techniques, this paper provides a new approach and method for the automation of ASD diagnosis.

## 2 Method

Our algorithm primarily operates by learning an autoencoder that takes MRI images as input. This autoencoder is then used for reconstruction, and the reconstructed MRI images are fed into a convolutional neural network, where a classifier determines the clinical validity of the images. This section analyzes both the autoencoder model (see Section 2.1) and the classifier model (see Section 2.2) proposed by our method.

### 2.1 Autoencoder model architecture

#### 2.1.1 Model architecture

**Figure 1:**
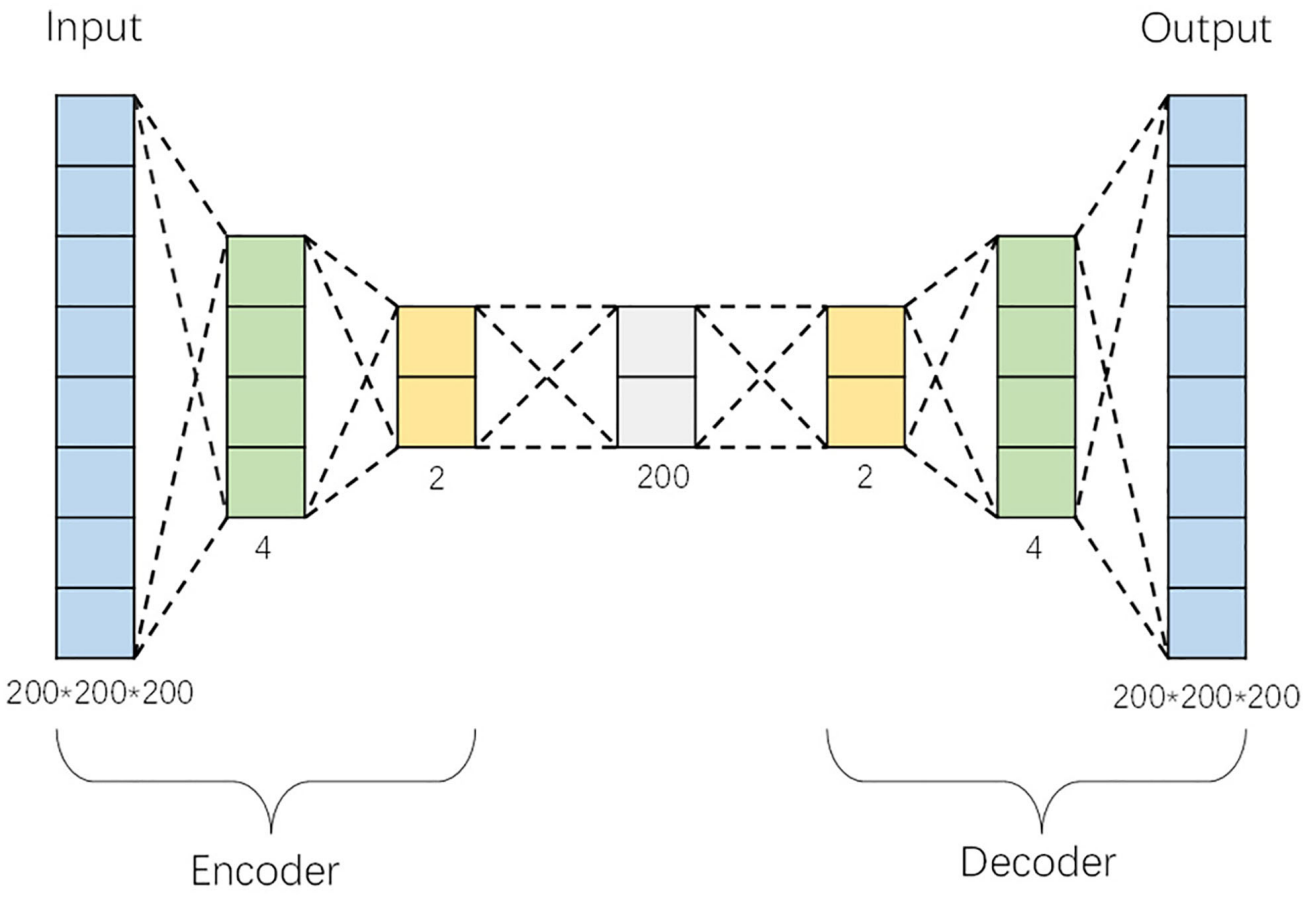
One-dimensional autoencoder architecture.

The autoencoder is composed of an encoder and a decoder. **Figure 1** displays the structure of this one-dimensional autoencoder, which utilizes minimal encoders, decoders, and hidden layers to simplify the model while achieving high reconstruction performance. The autoencoder starts with an input layer, followed by the encoder, which transforms the input data into feature vectors through two fully connected layers. Specifically, the encoder contains two ReLU-activated dense layers with 4 and 2 units, respectively, which compress the input data into a compact feature representation. An intermediate fully connected layer is situated between the encoder and decoder. One fully connected layer is located between the encoder and decoder (model hyperparameters optimized using Bayesian hyperparameter optimization algorithm). The intermediate hidden layer adopts the settings from the literature (21), and the data serves as input for the decoder. The decoder mirrors the encoder’s structure, consisting of two ReLU-activated dense layers with 2 and 4 units, respectively. The final layer of the decoder features a tanh activation function and has an output size of (8000000), which corresponds to the flattened three-dimensional image data. This symmetry between the encoder and decoder layers facilitates accurate reconstruction of the original input data.

The overall model adopts a symmetrical structure, with the output of each layer serving as the input for the next, thereby facilitating layer-by-layer training and effective learning of data representations. For training the autoencoder, we utilized the Adam optimizer, with a learning rate of 0.001 and an epsilon value of 1e-08. The Adam optimizer is well-suited for training large-scale and high-dimensional data due to its adaptive learning rate capabilities, which adjust based on gradient statistics and improve convergence during the training process.

#### 2.1.2 Algorithm overview

Assuming the input data is y and the output data is x, the encoding and decoding process of the autoencoder can be described as (22):


(1) $$h_{1}=\sigma_{e}\left(W_{1}x+b_{1}\right)$$

(2) $$\text{y}=\sigma_{d}\left(W_{2}h_{1}+b_{2}\right)$$

Where *W*_1_ and *b*_1_ represent the encoding weights and biases, *W*_2_ and *b*_2_ represent the decoding weights and biases, *σ_e_* is a non-linear transformation, and *σ_d_* is the same non-linear transformation used during the encoding process.

#### 2.1.3 Loss function

The loss function is calculated using mean squared error loss:


(3) $$J\left(\text{W},\text{b}\right)=\sum \left(L\left(\text{x},\text{y}\right)\right)=\sum {\left||\text{y}-\text{x}|\right|}_{2}^{2}$$

Using gradient descent, the backpropagation algorithm updates parameters to minimize the error function. The update formulas for the weights *W* and biases *b* are as follows, where *v* is the learning rate:


(4) $$\text{W}=\text{W}-v\frac{\partial J\left(\text{W},\text{b}\right)}{\partial \text{W}}$$

(5) $$\text{b}=\text{b}-v\frac{\partial J\left(\text{W},\text{b}\right)}{\partial \text{b}}$$

For images, especially medical images that contain detailed pathological information, it is necessary to extract this information from the images as a whole. Since autoencoders learn holistic representations and can effectively extract high-level abstract features, they have the capability to generate brain MRI images as needed.

### 2.2 CNN-based MRI classification discrimination model architecture

In this study, MRI identification is a binary classification problem, aimed at distinguishing whether a three-dimensional MRI belongs to a diseased (label: 1) or healthy (label: 0) category. An MRI dataset is reshaped into an array Tm×a×b (in this study, 128×128×128), serving as the input for the 3D CNN.

The designed convolutional neural network structure of our algorithm (partially detailed in **Supplementary Materials**) consists of convolutional layers, pooling layers, activation function layers, and fully connected layers, each performing specific functions. It begins with initial layers employing 3x3x3 filters: 2 filters in the first layer and 8 filters in subsequent layers with a stride of 2 for downsampling. Each convolutional layer uses ReLU activation. Batch normalization is applied for stability after select convolutional layers. Average pooling with a 2x2x2 kernel and stride of 2 follows, reducing spatial dimensions while retaining important features. Deeper layers increase complexity with 16, 32, 64, and 128 filters, maintaining spatial resolution. All use ReLU activation and batch normalization. Output from convolutional layers is flattened for fully connected layers. Two dense layers follow with 128 and 64 neurons, using ReLU and tanh activation. Dropout with a 0.5 rate is applied after each dense layer to prevent overfitting. The final layer consists of 2 neurons with softmax activation for binary classification. Adam optimizer minimizes binary cross-entropy loss, assessing accuracy metrics. The final output of the 3D CNN is the probability values for the two classification labels, summing to one.

This study uses the Adam optimizer to optimize the 3D CNN, with a learning rate set at 0.00001 and includes a learning rate decay callback function, ReduceLROnPlateau, which dynamically reduces the learning rate. The initialization strategy follows the settings described in the referenced literature (23). Post-training, the model reports performance on the validation set (or new MRI).

## 3 Results

This section delineates the experimental framework and dataset used (refer to Section 3.1). We carried out a series of experiments to assess the efficacy of the proposed method through both qualitative (Section 3.2) and quantitative analyses (Section 3.3) of the results. Our testing setup was configured on a Windows 10 system, employing Python 3.6 and TensorFlow 2.5, with GPU acceleration provided by an NVIDIA GeForce GTX 1650 graphics card, which has 4.0GB of GPU memory. The source code and implementation details are publicly available at (https://github.com/Lyrae17514/Generation-and-Discrimination-of-Autism-MRI-Images-Based-on-Autoencoder).

### 3.1 Data description

The ABIDE I dataset, consisting of data from 17 international sites and encompassing 1112 subjects, was utilized in this study. Specifically, we employed data from 871 ABIDE I subjects that underwent quality checks, aligning with the dataset used by Mostafa et al. (20). This subset includes 403 subjects diagnosed with Autism Spectrum Disorder (ASD) and 468 from a typical control group (24). The subjects ranged from 7 to 64 years in age, and our study focused exclusively on the T1 dataset.

### 3.2 Qualitative analysis

#### 3.2.1 Generative performance evaluation experiment

**Figure 2:**
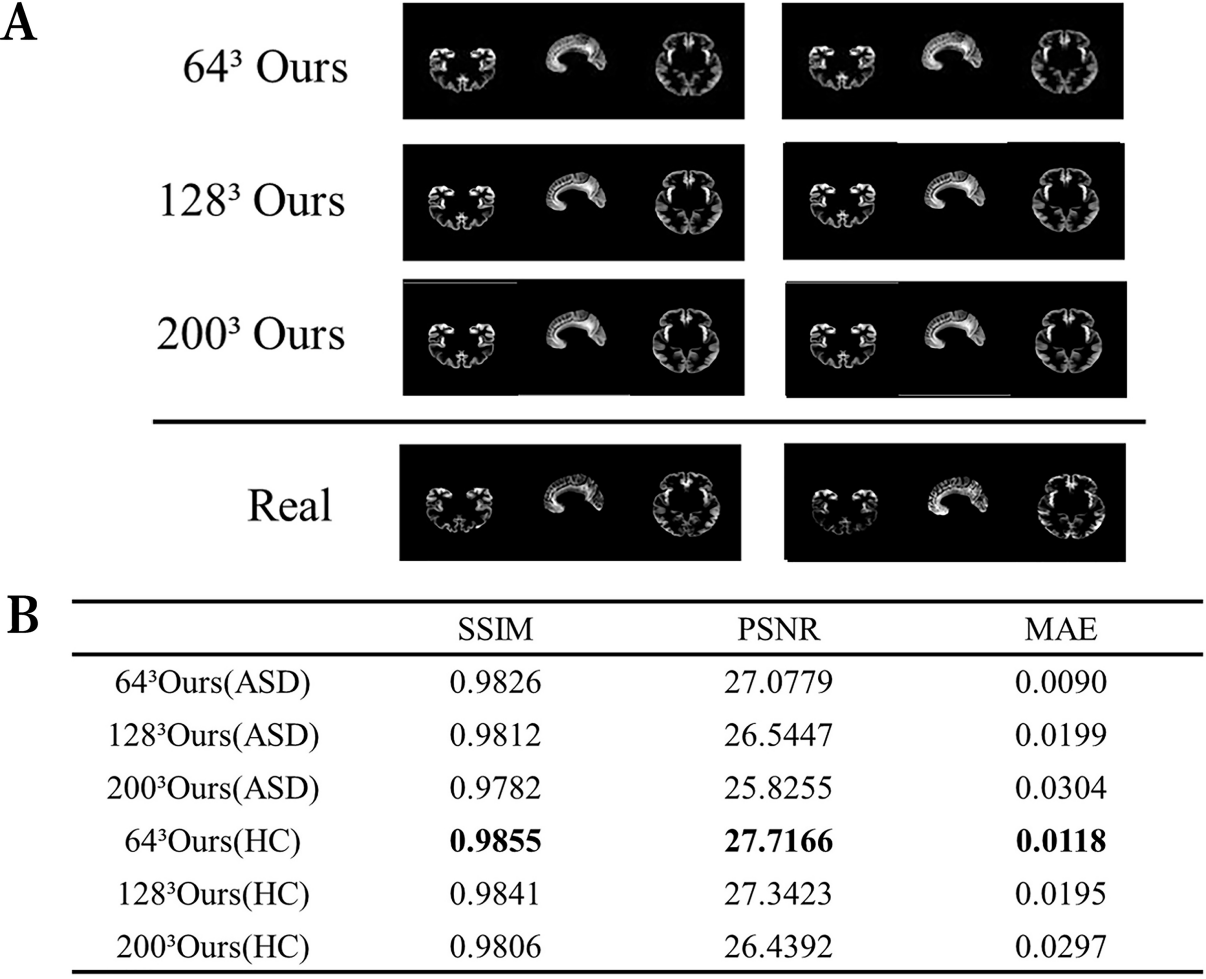
Visualization of the reconstructed image performance of the model. **(A)** Visualization of the generated image results. **(B)** Image reconstruction performance of ASD and HC. ASD represents the use of data from patients with ASD, while HC represents the healthy control group.

This experiment aimed to appraise the image reconstruction capabilities of the autoencoder. We assessed the image reconstruction efficacy of the autoencoder by inputting images of various sizes and comparing the output to the original images. Given the inclusion of critical pathological information within medical images, it is crucial to enhance the fidelity between the reconstructed and original images to preserve essential pathological structures. **Figure 2A** exhibits a comparative analysis of reconstructed images with dimensions of 64^3^, 128^3^, and 200^3^ against the originals, clearly demonstrating the autoencoder’s ability to accurately replicate details. **Figure 2B** further presents various performance metrics associated with the reconstructed images.

The data for this experiment comprised 3D data sourced from ANTS. The autoencoder employed mean squared error (MSE) as the loss function. Given the constraints imposed by the limited GPU memory capacity, our training methodology had to be specifically tailored to handle high-dimensional MRI data within these hardware limitations. The MRI data sets, characterized by their substantial memory footprint, necessitated a training approach that accommodates the GPU’s memory constraints. Consequently, the generator function was designed to dynamically select a single MRI image from the dataset at a time, resulting in a batch size of 1 for each training iteration. However, it also poses challenges such as increased training variability and slower convergence rates. To mitigate these issues and enhance training efficiency, the model employs the Adam optimizer, with a learning rate set at 0.001, known for its adaptive learning rate adjustments, which help in navigating the stochastic nature of the training process and improving convergence over extensive epochs. For instance, when generating MRI images of size 200^3^, the autoencoder can achieve its best SSIM score upon reaching 68000 epochs. When the autoencoder processes a 3D matrix of size 128^3^, it is flattened into a 1D array of length (2097152) as input. Both the encoding and decoding phases featured two fully connected layers, with neuron configurations of 4 and 2 (encoding) and 2 and 4 (decoding) respectively.

In the comparison between generated image results and real images (**Figure 2**), the generated images show no significant distortions or artifacts, effectively removing noise. This indicates that the model fits the data well without overfitting. Specifically, this means that the model demonstrates good adaptability to the training data. The reconstruction performance is high for images of various sizes, demonstrating the model’s robustness. Although some details in the generated images might appear slightly blurry, the overall quality remains high, likely reflecting the intrinsic characteristics of the autoencoder. Moreover, high values of Structural Similarity Index (SSIM) and Peak Signal-to-Noise Ratio (PSNR), coupled with low Mean Absolute Error (MAE), corroborate the autoencoder’s ability to generate high-quality brain MRI images.

#### 3.2.2 Classification performance evaluation experiment

The ROC curve is used to evaluate the classification and detection results. The curve closer to the top-left corner indicates a higher true positive rate and lower false positive rate, suggesting better classification ability of the model. The area under the ROC curve (AUC) is used to measure the performance. The classifier is validated on the validation set, and the ROC curve is plotted. The model has an average accuracy of 58.85%, with an accuracy of 63% in detecting healthy samples. The calculated AUC value is 0.60, indicating relatively good performance of the binary classifier with some predictive capability. Overall, this classifier can effectively screen ASD patients and healthy controls to a certain extent.

### 3.3 Quantitative analysis

In this section, we evaluate the reconstruction effectiveness of the quantized autoencoder and compare it with other models using the Structural Similarity Index (SSIM) (25) and Peak Signal-to-Noise Ratio (PSNR) to assess the generated images. **Table 1** presents the quantitative results of these evaluation metrics, with some model results referenced from Mostafa (20). The model studied in this paper has relatively high SSIM scores, indicating that the distribution of the generated samples closely resembles the distribution of real data. For the 128^2^ resolution, the model in this study achieved excellent SSIM scores, and it also performed well at a resolution of 256^2^. The generated images have high SSIM and PSNR values, as well as low MAE (Mean Absolute Error), showcasing excellent image quality and accurate pixel matching.

**Table 1:** Comparison of quantitative results.

	SSIM↑	PSNR↑	MSE↓
CAE	0.22	0.79	0.21
128^2^ Ours	0.58	19.42	0.01
256^2^ Ours	0.62	19.91	0.01

The upward arrow indicates that a higher score is better, meaning the image is closer to the real image. Conversely, the downward arrow signifies that a lower score is preferable, indicating that the image error is smaller.

To ensure a fair comparison with Mostafa’s proposed CAE model, we maintained consistency in data processing, using T1-weighted MRI slice images of the healthy control group for autoencoder reconstruction (26). When generating images at sizes of 256^2^ and 128^2^, the autoencoder adjusted the size of the input and output images and only modified the structure of the intermediate hidden layer of the autoencoder.

Compared to Mostafa’s proposed CAE model, the autoencoder proposed in this paper exhibits superior SSIM scores at a size of 128^2^. When generating larger images at a size of 256^2^, the PSNR metric still performs relatively well. The higher SSIM and PSNR scores, along with the lower MSE, indicate that the generated images preserve more structural details and have lower noise levels. This suggests that our autoencoder produces reconstructed images with higher clarity and quality while preserving the original information. Therefore, this experiment demonstrates that our autoencoder model generates images that are closer to the original images, exhibiting good quality and excellent generalization capabilities.

## 4 Discussion

First, this section analyzed the computational complexity of the proposed method (see section 4.1). Second, the generator interpretation was discussed in section 4.2. Third, the influence of optimizers on performance was discussed (see Section 4.3).

### 4.1 Computational complexity

The autoencoder proposed in this study is based on a fully connected neural network structure. The complexity of this model can be estimated by calculating the number of parameters, which is proportional to the number of connection weights between the input and output layers, as well as the number of bias terms. The time complexity calculation is as follows:


(6) $$O\left(U\alpha \right)=O\left(\sum_{\alpha =1}^{d}n_{\alpha -1}\cdot n_{\alpha }+n_{\alpha }\right)$$

Where *n_α-1_* and *n_α_* represent the number of neurons in the (*α*-1) and *α* layers, respectively. Assuming *α* represents the number of layers and *U* represents the number of neurons per layer, the time complexity of the autoencoder model is as described above.

### 4.2 Interpretation of autoencoders for MRI generation

This set of experiments aims to elucidate the performance of autoencoders in generating MRI images. The generator proficiently learns the correlations and distribution patterns among various features from the training dataset, successfully capturing the essential characteristics of the images and reconstructing them based on these identified patterns. We visualize color feature mapping of some brain regions to reflect the feature correlations between the generated images and the original images.

We train the model using ASD patient data and provide color mapping of generated image slices and real sample slices to demonstrate the model’s performance. Both generated and real sample slices are aligned in the same direction. The resultant grayscale images are then transformed into color-mapped images, where different colors denote the intensities of various features.

**Figure 3:**
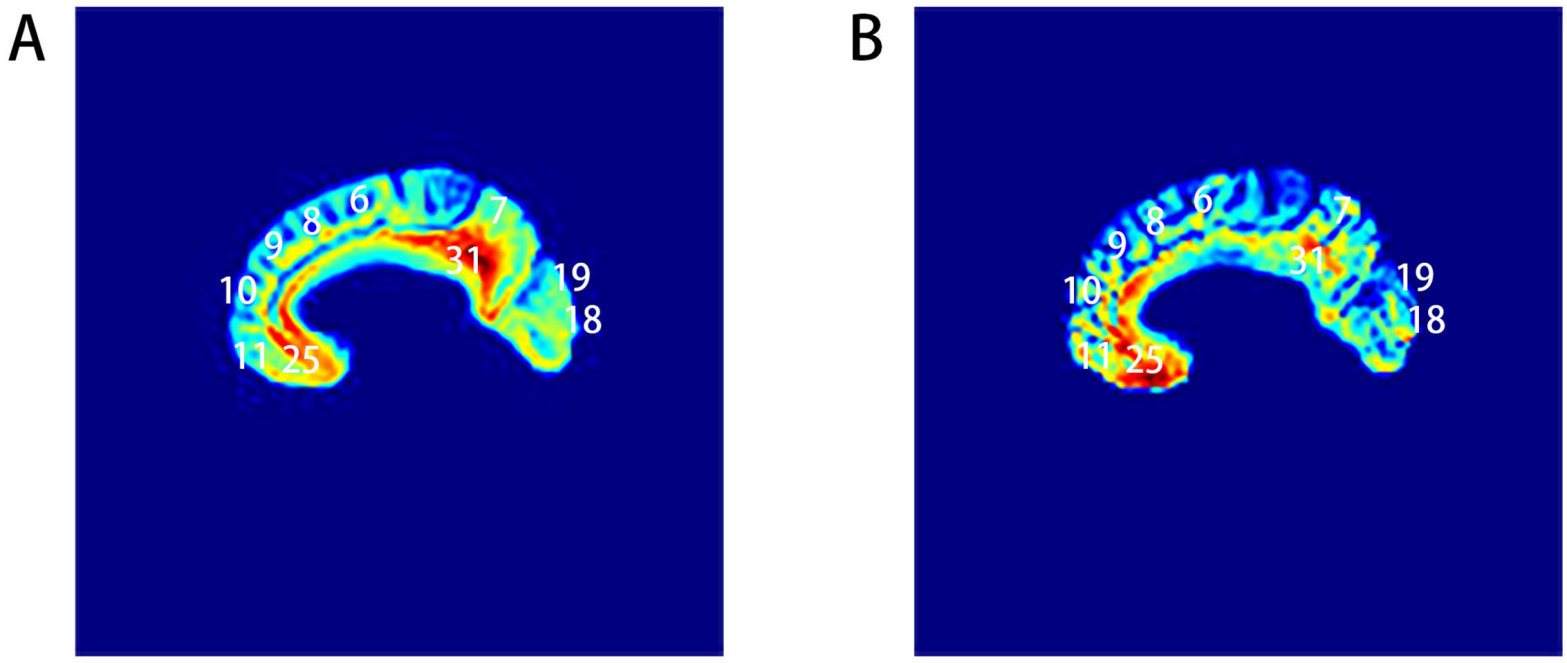
Visualizations of feature mapping, including **(A)** generated samples and **(B)** real samples.

**Figure 3** presents 2D brain MRI feature mapping slices, displaying partial Brodmann areas. In **Figure 3**, A represents the color mapping of the generated image slice, while B represents the color mapping of the real sample slice, with yellow representing higher intensity and blue representing lower intensity. The similar color distributions indicate a high degree of feature correspondence and an enhancement of information during the generation process. Notably, in parts of Brodmann areas 7, Brodmann areas 9, and Brodmann areas 18, there is an increase in feature intensity, signifying enhanced feature information, suggesting that the autoencoder primarily generates images by correctly extracting and processing features from these areas. This result is consistent with partial research findings of Bhaumik (2) and Donato (3). In summary, the autoencoder model is capable of accurately extracting and reflecting the features hidden within MRI images for image generation.

### 4.3 The influence of optimizer on performance

This subsection compares different optimization methods in the autoencoder, including momentum SGD, RMSprop, Adagrad, and Adam. Corresponding image have been added to the **Supplementary Materials**. In this study, RMSprop and Adam display superior performance, while Adagrad and momentum SGD are somewhat less effective. Adagrad and SGD have relatively fixed learning rates. Fixed learning rates may fail to effectively train models when there are significant differences in learning rate requirements across layers or parameters, particularly in complex optimization scenarios, leading to poor convergence or slow training speeds that impact performance. In contrast, RMSprop and Adam provide smooth and stable parameter updates, which help in avoiding drastic fluctuations in learning rates and thereby facilitate more effective training. RMSprop and Adam optimizers are generally more suitable for handling complex, non-convex optimization problems due to their ability to adapt to varying learning rate requirements, sparse gradients, and complex loss surfaces. SGD and Adagrad optimization methods did not succeed in achieving fast training of neural networks with complex structures as anticipated. The primary reason is likely the different adaptability of these optimizers in handling specific model gradient updates. In contrast, RMSprop and Adam, with their adaptive learning rate adjustment capabilities, typically perform more effectively in dealing with complex model structures and gradient characteristics.

## 5 Conclusion and future work

This study proposes a method based on autoencoders for generating brain magnetic resonance imaging (MRI) images of patients with Autism Spectrum Disorder (ASD) and non-ASD individuals, and for discriminating ASD based on the generated images. Through experimental validation, we found that the proposed autoencoder model can effectively generate diverse MRI images of both ASD and non-ASD patients, capturing the structural differences in the brains of the patients. Additionally, our designed ASD classifier based on the generated images demonstrates good performance in the task of ASD discrimination, showing better robustness and generalizability compared to traditional classification methods based on original MRI images. These results provide new insights and approaches for the application of deep learning technologies in ASD research, with the potential to contribute to the automation of ASD diagnosis and research. However, generating MRI images of ASD and non-ASD patients using autoencoders faces certain limitations, such as restricted image sizes due to the computational complexity of MRI data, currently preventing the flexible generation of MRI images in various sizes. Future work may enhance model performance, expand datasets, and explore additional methods for medical image analysis based on automated image generation, thereby promoting early diagnosis and personalized treatment of ASD. Additionally, while this study assesses the clinical validity of the generated images using classifiers, future research should integrate pathophysiology to more deeply analyze the clinical application potential of the model, thus enhancing its supportive role in ASD treatment.

## Data Availability

The original contributions presented in the study are included in the article/**Supplementary Material**. Further inquiries can be directed to the corresponding authors.
