# Supplementary material for: Generation and discrimination of autism MRI images based on autoencoder
Source: Front Psychiatry. 2024 Oct 14;15:1395243. doi: 10.3389/fpsyt.2024.1395243 (PMC11521099; doi:10.3389/fpsyt.2024.1395243)
Supplement: Supplementary file 1 [file DataSheet1.pdf]

Supplementary Material

1 SUPPLEMENTARY DATA

2 SUPPLEMENTARY TABLES AND FIGURES

2.1 Figures

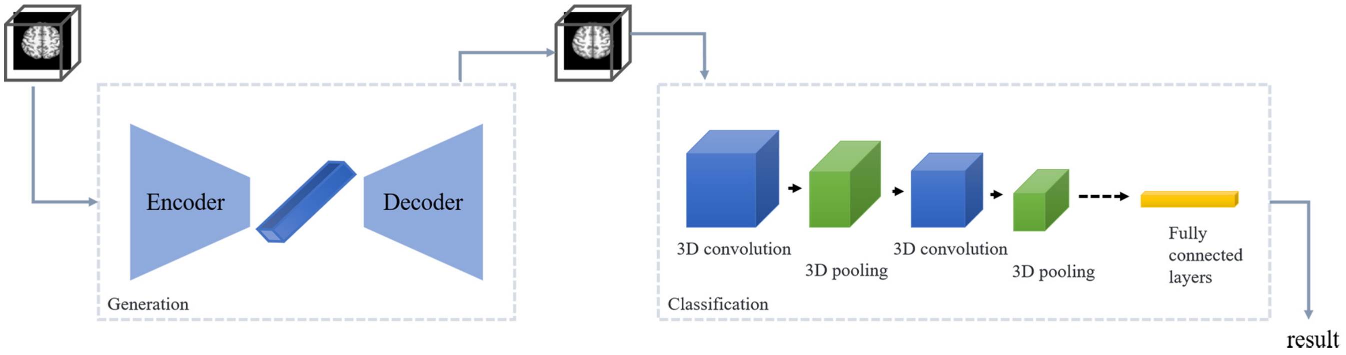

Figure S1. Flowchart

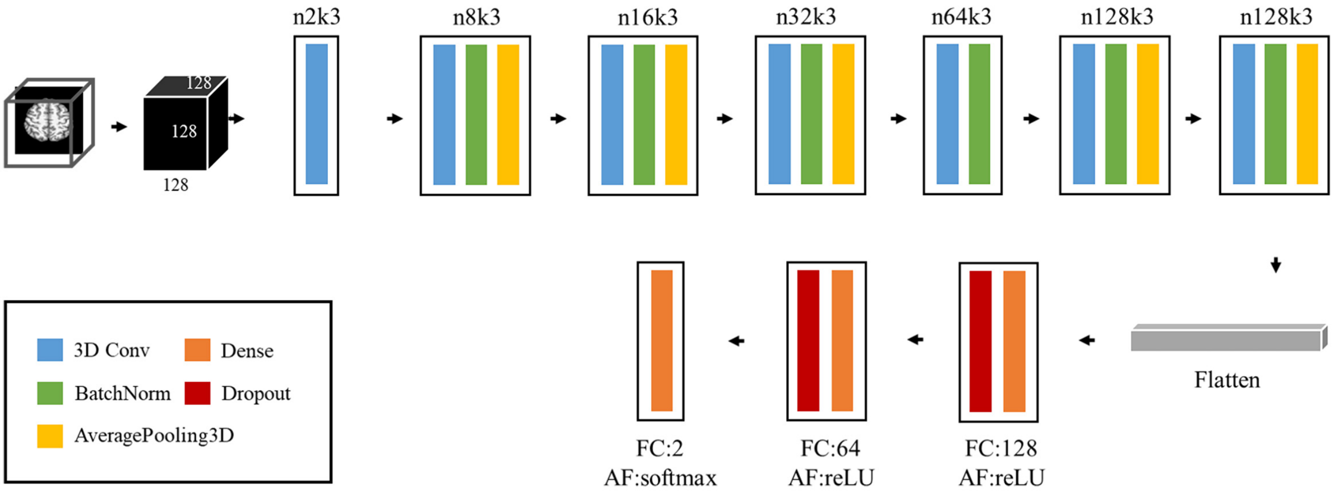

Figure S2. CNN architecture diagram

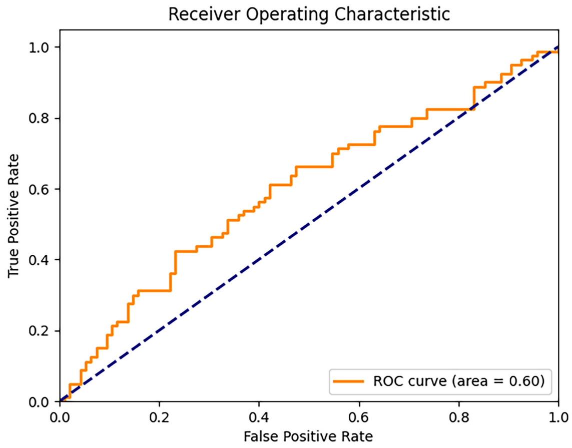

Figure S3. ROC curve of the classifier

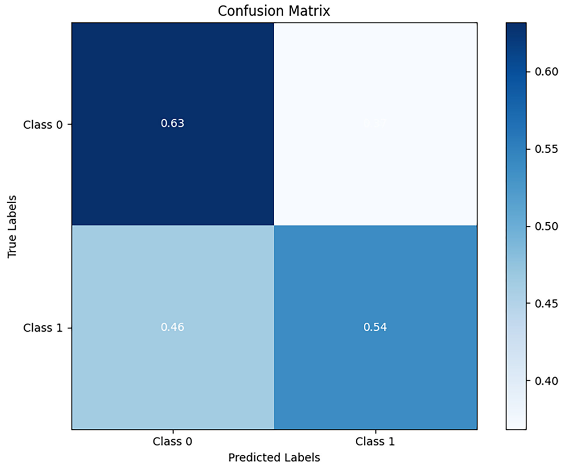

Figure S4. Confusion Matrix of the Classifier

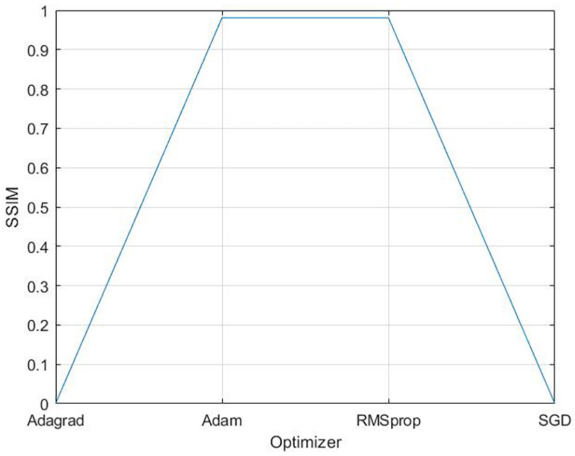

Figure S5. Comparison of candidate optimization methods.
